# Realization of Large Low-Stress Elastocaloric Effect in TiZrNbAl Alloy

**DOI:** 10.3390/ma17040885

**Published:** 2024-02-14

**Authors:** Bang-He Lv, Hua-You Xiang, Shang Gao, Yan-Xin Guo, Jin-Han Yang, Nai-Fu Zou, Xiaoli Zhao, Zongbin Li, Bo Yang, Nan Jia, Hai-Le Yan, Liang Zuo

**Affiliations:** 1 Key Laboratory for Anisotropy and Texture of Materials (Ministry of Education), School of Material Science and Engineering, Northeastern University, Shenyang 110819, Chinazhaoxl@mail.neu.edu.cn (X.Z.); lizb@atm.neu.edu.cn (Z.L.); lzuo@mail.neu.edu.cn (L.Z.); 2 Institute of Materials Science and Engineering, Shenyang Aerospace University, Shenyang 110136, China

**Keywords:** shape memory alloy, elastocaloric effect, TiZrNbAl, superelasticity, solid-state refrigeration

## Abstract

Seeking novel high-performance elastocaloric materials with low critical stress plays a crucial role in advancing the development of elastocaloric refrigeration technology. Here, as a first attempt, the elastocaloric effect of TiZrNbAl shape memory alloy at both room temperature and finite temperatures ranging from 245 K to 405 K, is studied systematically. Composition optimization shows that Ti-19Zr-14Nb-1Al (at.%), possessing excellent room-temperature superelasticity with a critical stress of around 100 MPa and a small stress hysteresis of around 70 MPa and outstanding fracture resistance with a compressive strain of 20% and stress of 1.7 GPa, demonstrates a substantial advantage as an elastocaloric refrigerant. At room temperature, a large adiabatic temperature change ($\Delta T_{\mathrm{ad}}$) of −6.7 K is detected, which is comparable to the highest value reported in the Ti-based alloys. A high elastocaloric cyclic stability, with almost no degradation of $\Delta T_{\mathrm{ad}}$ after 4000 cycles, is observed. Furthermore, the sizeable elastocaloric effect can be steadily expanded from 255 K to 395 K with a temperature window of as large as 140 K. A maximum $\Delta T_{\mathrm{ad}}$ of −7.9 K appears at 355 K. The present work demonstrates a promising potential of TiZrNbAl as a low critical stress and low hysteresis elastocaloric refrigerant.

## 1. Introduction

The conventional vapor-compression refrigeration method, employing ozone-depleting substances like hydrofluorocarbons (HFC) and hydrochlorofluorocarbons (HCFC) as refrigerants, has been recognized as a notable factor in environmental harm and climate change [1,2,3]. Therefore, it is imperative to innovate and establish alternative, environmentally friendly refrigeration technologies. With its high Carnot efficiency and cost-effectiveness, as highlighted in studies [4,5,6], elastocaloric solid-state refrigeration emerges as a promising option due to the release and absorption of latent heat during stress-induced reversible structural transitions [7]. Nowadays, a primary obstacle hindering the advancement of elastocaloric refrigeration methods lies in the design or identification of elastocaloric refrigerants with superior performance.

Due to the large caloric effects and highly efficient heat transfer, shape memory alloys (SMAs) currently stand out as the most promising elastocaloric refrigerant [8,9,10], including Ni-Ti-based [11,12,13,14,15], Cu-based [16,17,18], Fe-based [19,20,21], and Heusler-type [22,23,24,25,26,27,28,29] alloys. Despite the enormous potential exhibited by these alloys, there is still considerable room for improvement. For example, Ni-Ti [11,12,13,14] and Ni-Mn-Ti [22,23,24] display a giant adiabatic temperature change $\Delta T_{\mathrm{ad}}$ (∼30 K) but suffer from high critical stress $\sigma_{\mathrm{cr}}$ (250∼700 MPa) and large stress hysteresis $\Delta \sigma_{\mathrm{hys}}$ (160∼350 MPa) across martensitic transformation. These issues can be addressed in Cu-based [16,17,18] and NiMn-based [25,28,30] and NiFeGa-based Heusler-type alloys [27,28,29], but the inherent brittleness of these alloys introduces a new challenge [28]. Considering practical applications, an optimal elastocaloric refrigerant should exhibit high $\Delta T_{\mathrm{ad}}$, low $\sigma_{\mathrm{cr}}$, and $\Delta \sigma_{\mathrm{hys}}$, alongside excellent mechanical properties and high cyclic stability [31]. However, as far as our knowledge extends, current elastocaloric refrigerants cannot simultaneously meet these requirements. Hence, there is significant value in exploring novel high-performance elastocaloric refrigerants.

Recently, the elastocaloric effect (eCE) of β-type Ti-based SMAs has been gaining increasing attention owing to their excellent mechanical properties, narrow stress hysteresis, and broad working temperature range [32,33,34,35]. The eCE of Ti-based SMAs originates from the stress-induced martensitic transformation from body-centered cubic austenite (β phase) to the orthorhombic martensite ($\alpha^{\prime \prime }$ phase) [36,37,38]. TiZrNbAl, with compositions of Ti-18Zr-(12-16)Nb-(0-4)Al (at.%), is a kind of high-performance Ti-based SMA characterized by outstanding mechanical properties and superelasticity [39,40], but its elastocaloric performance has not been explored. In this context, the room temperature and temperature-dependent eCE of TiZrNbAl SMAs are studied systematically in this work. Three alloys with nominal compositions of Ti-19Zr-14Nb-(1,2,3)Al (at.%, termed as 1Al, 2Al and 3Al) are fabricated. The reason for increasing the content Zr content (from 18 at.% to 19 at.%) is to enhance the phase stability of the β phase [37,38], aiming to obtain a single β austenite phase at room temperature. Variation of the Al content from 1 at.% to 3 at.% is aimed at achieving a superior superelastic and elastocaloric performance. The crystal structures and the behaviors of the heat- and stress-induced martensitic transformation of the 1Al, 2Al, and 3Al alloys are first examined (Section 3.1). Then, the microstructure and fracture resistance of the 1Al alloy, which demonstrates a substantial advantage of superelasticity against the other two alloys, is characterized (Section 3.2). Lastly, $\Delta T_{\mathrm{ad}}$ of eCE, cyclic stability, and the temperature dependence of eCE at temperatures ranging from 245 K to 405 K of the 1Al alloy are investigated (Section 3.3).

## 2. Experimental Details

Master ingots with nominal compositions of Ti-19Zr-14Nb-(1,2,3)Al (at.%) were prepared by arc-melting technique under the Ar atmosphere using high-purity elements of Ti, Zr, Nb and Al (>99.98 wt%). All raw high-purity metals were purchased from Shenyang Jiabei Trading Co., Ltd., Shenyang, China. To ensure a good chemical composition homogeneity, the ingots were flipped over and remelted six times. Afterward, the as-casted ingots were sealed into a quartz tube filled with the Ar atmosphere and then homogenized at 1273 K for 2 h, followed by rapid quenching into ice water. Using energy-dispersive spectrometry (EDS), we found that the actual compositions of Ti-19Zr-14Nb-*x*Al (at.%) with *x* = 1, 2, and 3 were determined to be Ti_65.66_Zr_19.24_Nb_14.18_Al_0.92_ (at.%), Ti_64.80_Zr_19.15_Nb_14.17_Al_1.88_ (at.%) and Ti_64.19_Zr_18.86_Nb_14.10_Al_2.85_ (at.%), respectively, aligning well with their nominal compositions. The critical temperatures of martensitic transformation were determined by the differential scanning calorimeter (DSC, TA-Q100) with a heating/cooling rate of 10 K min^−^^1^. The room-temperature crystal structure was examined using X-ray diffraction (XRD, Rigaku Smartlab, Tokyo, Japan) with Cu-*K*α radiation. The microstructure was characterized by the scanning electron microscope (SEM, JSM 7001F) with an electron backscatter diffraction (EBSD) camera. The mechanical properties and temperature-dependent elastocaloric effect were measured by a universal testing machine (Shimadzu AG-XPlus 50 kN) equipped with a temperature control attachment. Rectangular specimens with a dimension of 3×4×6 mm^3^ were used for compression tests. For each alloy, the measurements are performed on at least three samples. The strain was traced using the digital image correlation (DIC) (VIC-3D9, Correlated Solutions, Irmo, SC, USA) technique. $\Delta T_{\mathrm{ad}}$ was directly recorded using a Pt100 resistance temperature detector pegged at the center of the sample surface.

## 3. Results and Discussion

### 3.1. Composition Design

**Figure 1:**
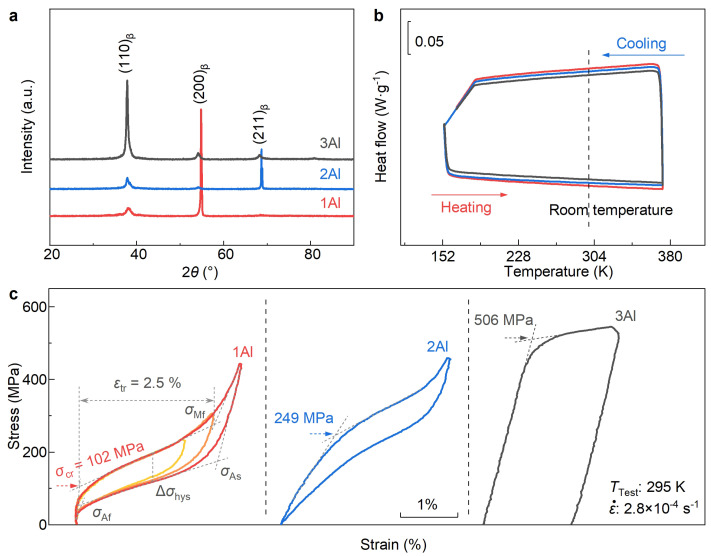
Crystal structure, martensitic transformation and superelasticity. (**a**) Room-temperature XRD patterns of the bulk samples; (**b**) DSC curves; (**c**) Compressive stress–strain curves measured at room temperature. 1Al, 2Al and 3Al represent the Ti-19Zr-14Nb-*x*Al (at.%) alloy for *x* = 1, 2, and 3, respectively.

In Figure 1a, the X-ray diffraction patterns of the 1Al, 2Al, and 3Al bulk samples at room temperature are presented. It is evident that all the studied alloys exhibit a single body-centered cubic (BCC) phase (i.e., the β phase) with a space group of Im$\bar{3}$m at room temperature. The discrepancy in the relative intensity of different diffraction peaks among these alloys may be attributed to their distinct crystallographic preferred orientations in the examined bulk samples. Figure 1b displays the DSC curves of the 1Al, 2Al, and 3Al alloys measured across temperatures ranging from 150 K to 370 K at a heat/cooling rate of 10 K min^−1^. Notably, during the heating and cooling processes, no significant endothermic or exothermic peaks, which are key indicators of first-order martensitic transformation [26], are detected. This result suggests that the structural transition, which is the origin of elastocaloric effect (eCE), cannot be driven by the temperature field in the studied alloys. A similar phenomenon has also been observed in other reported Ti-based elastocaloric refrigerants [33,34,35].

Figure 1c shows the cyclic compressive stress–strain curves of the 1Al, 2Al, and 3Al alloys measured at 295 K. For all samples, a widely employed mechanical training method involving ten loading–unloading cycles is performed before each measurement to achieve stable stress–strain behavior. The stress–strain curve gradually stabilizes with the progression of mechanical training after several loading–unloading cycles (Supplementary Materials Figure S1, see also references [41,42,43,44] therein), consistent with previous observations in Ti-based SMAs [42,45]. For the 1Al and 2Al alloys, excellent superelasticity behavior in a fully reversible manner is observed, with a maximum strain of 3%. This result suggests that, despite the absence of thermal-induced structural transition (Figure 1b) in the 1Al and 2Al alloys, the martensitic transformation can be triggered by an external uniaxial mechanical field. Thus, both the 1Al and 2Al alloys demonstrate potential as elastocaloric refrigerants.

By the tangent method, the critical stress $\sigma_{\mathrm{cr}}$ of the 1Al and 2Al alloys is measured at 102 MPa and 249 MPa, respectively. Clearly, $\sigma_{\mathrm{cr}}$ shows an elevating tendency with the increase of Al content. This observation is in good agreement with the investigation by H. Y. Kim and coworkers that the addition of Al in the TiZrNb alloys tends to stabilize the β phase [39]. For both samples, the stress hysteresis $\Delta \sigma_{\mathrm{hys}}$, defined by $(\sigma_{\mathrm{cr}}+\sigma_{\mathrm{Mf}}-\sigma_{\mathrm{As}}-\sigma_{\mathrm{Af}})/2$, where $\sigma_{\mathrm{cr}}$, $\sigma_{\mathrm{Mf}}$, $\sigma_{\mathrm{As}}$ and $\sigma_{\mathrm{Af}}$ represent the onset and the end of forward and inverse martensitic transformation, respectively, is measured to be around 70 MPa. This value is lower than NiMnTi (100∼200 MPa) [22,24], NiTi-based (100∼300 MPa) [11,12,13,14,46] and comparable to typical low-stress hysteresis SMAs, such as Cu-based (∼60 MPa) [16,17], and NiFeGa-based alloys (∼50 MPa) [27,29,47]. The low $\Delta \sigma_{\mathrm{hys}}$ is beneficial for reducing energy dissipation and improving functional stability.

Unlike the 1Al and 2Al alloys, the 3Al alloy does not exhibit superelasticity. Instead, it demonstrates a typical irreversible plastic deformation stress–strain curve with a yielding stress of 506 MPa. The disappearance of superelasticity in the 3Al alloy can be attributed to the increased stability of the β phase due to the higher Al content. This leads to an elevated $\sigma_{\mathrm{cr}}$ requirement for initiating martensitic transformation, surpassing the threshold for nucleation and dislocation movement. Consequently, during the loading process, dislocation movement takes precedence over martensitic transformation. The above findings evidence that the Al element can greatly tailor the stability of the austenite phase in the metastable β-Ti alloys and further adjust the required critical driving force for superelasticity.

Compared with the 2Al and 3Al alloys, the 1Al alloy demonstrates an obvious advantage as an elastocaloric refrigerant, particularly attributed to its low $\sigma_{\mathrm{cr}}$ (around 100 MPa). This value is lower than NiTi-based (250∼700 MPa) [14,48,49,50] and NiMnTi (300∼450 MPa) [22,23,24], and comparable to the low driving force alloy system like Cu-based (110∼200 MPa) [16,51], NiMnIn-based (50∼150 MPa) [25,26,52], NiFeGa-based (50∼180 MPa) [27,28,29]. On the one hand, the low $\sigma_{\mathrm{cr}}$ supports the miniaturization of refrigeration equipment, paving the way for the application of micro-/nano-electronic device cooling [26,53]. On the other hand, when the driving force approaches the critical stress for plastic deformation, it becomes less conducive to the cyclic stability of elastocaloric refrigeration, owing to the increased likelihood of dislocation generation and accumulation during the mechanical loading [54]. Thus, in what follows, the attention is paid to the 1Al alloy.

### 3.2. Microstructure and Fracture Resistance

#### 3.2.1. Microstructure

**Figure 2:**
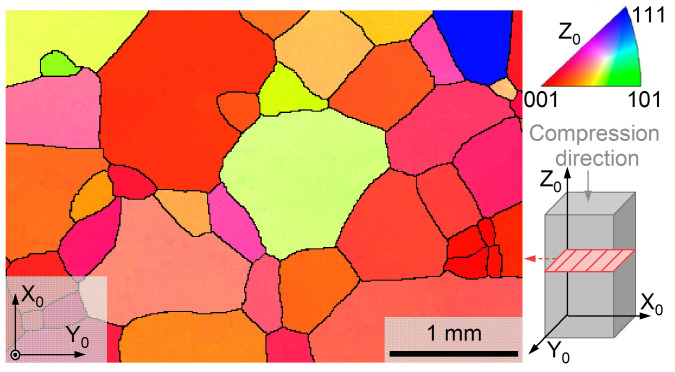
**Room-temperature crystallographic orientation micrograph of the 1Al alloy**. The color is rendered based on inverse pole figure (IPF) indices along Z_0_.

With the EBSD technique, the morphological and crystallographic features of the microstructure of the 1Al alloy are characterized. At room temperature, the sample is in the single-β austenite phase (Supplementary Materials Figure S2), in good agreement with the XRD result (Figure 1a). Figure 2 shows the crystallographic orientation maps of the 1Al alloy expressed by using inverse pole figure (IPF) indices along the Z_0_ direction of the sample. Notably, the sample is composed of coarse grains with an average grain size of around 500 μm. Generally, the large grain size is advantageous for enhancing the superelasticity and elastocaloric performance of shape memory alloys. In the fabricated sample, no obvious crystallographic preferred orientation (texture) exists, which is common in the arc-melting sample [55,56].

#### 3.2.2. Fracture Resistance

**Figure 3:**
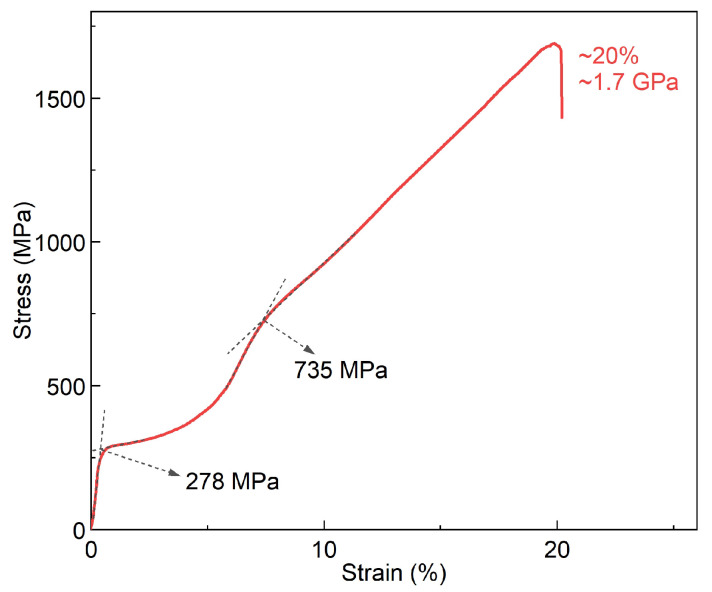
**Compressive fracture stress-strain curve of the 1Al alloy measured at room temperature**. A typical two-stage yield is observed.

Figure 3 shows the room-temperature compressive fracture stress–strain curve of the 1Al alloy measured with a strain rate of 0.28 × 10^−3^ s^−1^. Remarkably, the studied 1Al alloy exhibits outstanding fracture resistance, reaching a maximum of compressive stress $\sigma_{m}$ and strain $\varepsilon_{m}$ of ∼1.7 GPa and 20%, respectively. This performance is significantly superior to those of Heusler-type (Ni,Co)_2_(Fe,Mn)-based (0.5 GPa∼1.5 GPa, 6%∼18%) [57,58,59] CuAl(Mn,Ni)-based (0.8 GPa∼1.2 GPa, 7%∼13%) [60,61] refrigerants, and is comparable to that of NiTi [48,62]. Such excellent fracture resistance exhibited by the 1Al alloy is greatly desirable for achieving outstanding cyclic stability of the elastocaloric effect.

Notably, the stress–strain curve of the 1Al alloy is featured with typical two-stage yielding. The first yielding, with a $\sigma_{\mathrm{cr}}$ of 278 MPa, is associated with stress-induced martensitic transformation, while the second yielding, with a critical stress of 735 MPa could be attributed to the nucleation and movement of dislocation slip [36]. One may note that the critical stress of the first yielding in Figure 3 (278 MPa) is higher than that observed in the superelasticity measurement (102 MPa, Figure 1c). This difference arises because, in the superelasticity measurement, the sample undergoes mechanical training with ten loading–unloading cycles before the measurement, whereas no mechanical training is performed in the investigation of fracture resistance. As is known, mechanical training can greatly reduce $\sigma_{\mathrm{cr}}$ of stress-induced martensitic transformation in Ti-based shape memory alloys [42,45] (Supplementary Materials Figure S1, see also references [41,42,43,44] therein).

### 3.3. Elastocaloric Performance

#### 3.3.1. Adiabatic Temperature Change

**Figure 4:**
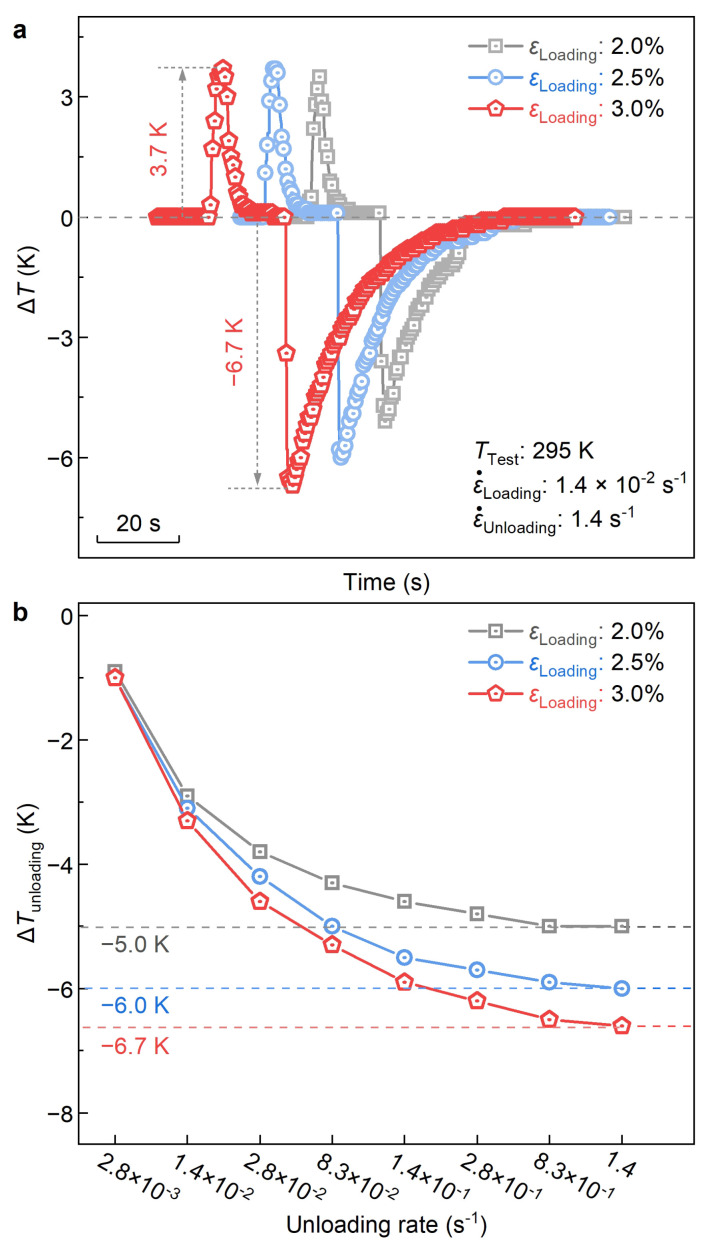
**Room-temperature elastocaloric effect of the 1Al alloy**. (**a**) Temperature change profiles under the applied strains of 2.0%, 2.5%, and 3.0%. (**b**) Strain rate dependences of adiabatic temperature change ($\Delta T_{\mathrm{ad}}$) during the unloading process.

Figure 4a depicts the temperature change profiles of the 1Al alloy during the compressive loading–unloading cycle with applied strains of 2.0%, 2.5%, and 3.0%. Herein, to assess the eCE potential of the studied alloy, a high unloading strain rate of 1.4 s^−1^ is employed to achieve an approximate adiabatic condition. At a strain of 3.0%, a large $\Delta T_{\mathrm{ad}}$ of −6.7 K is detected during the unloading process. This value stands out as one of the most outstanding performances among various Ti-based alloys, such as TiZrNbSn (−5.9 K at 303 K [33]), TiNbZrTa (−4.2 K at 298 K [63]), and TiZrCrSn (−6.5 K at 300 K [35]). Note that with the decrease of applied strain, the magnitude of $\Delta T_{\mathrm{ad}}$ gradually decreases. This phenomenon could be attributed to the reduced volume fraction during stress-induced martensitic transformation at a smaller applied strain [22].

In Figure 4b, we plot the strain-rate dependence of $\Delta T_{\mathrm{ad}}$ measured at the fixed applied strains of 2.0%, 2.5%, and 3.0%, respectively. For all the applied strain, with the increased strain rate, the magnitude of $\Delta T_{\mathrm{ad}}$ undergoes a sharp increase initially (at stain rates less than 8.3 × 10^−2^ s^−1^) and then tends to be stable (at strain rates higher than 8.3 × 10^−1^ s^−1^). This result shows that 1.0 s^−1^ could be used at the strain rate threshold for the elastocaloric measurement. $\Delta T_{\mathrm{ad}}$ measured with a strain rate larger than 1.0 s^−1^ could reflect the real elastocaloric performance. For the studied 1Al alloy, at the strains of 2.0%, 2.5%, and 3.0%, the equilibrium $\Delta T_{\mathrm{ad}}$ reach −5.0 K, −6.0 K, and −6.7 K, respectively.

#### 3.3.2. Cyclic Stability

**Figure 5:**
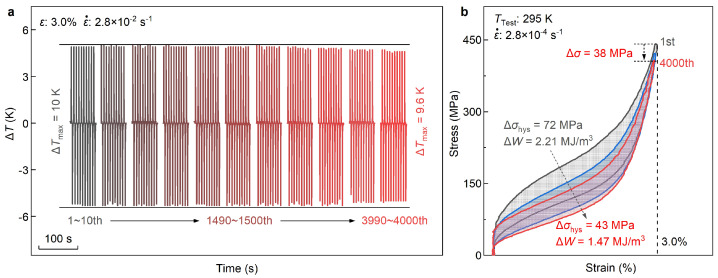
**Cyclic stability of elastocaloric effect of the 1Al alloy**. (**a**) Evolution of temperature change profile from the 1st to 4000th cycles. For clarity, the successive set of 11 profiles is displayed at an interval of every 500 cycles (for the first three sets, the interval is 50 cycles). The stain rate in both the loading and unloading process is 2.8 × 10^−^^2^ s^−^^1^. (**b**) Comparison of superelasticity stress–strain curves at the 1st, 1500th, and 4000th cycles.

The cyclic stability of the elastocaloric effect, a crucial factor in achieving practical elastocaloric refrigeration, is assessed by monitoring the variation of $\Delta T_{\mathrm{ad}}$ during continuous loading–unloading cycles. Figure 5a depicts the temperature change profiles of the 1Al alloy at several selected elastocaloric cycles, ranging from the 1st to 4000th cycle. Here, a compressive strain of 3% and a medium strain rate of 2.8 × 10^−^^2^ s^−^^1^ (for both loading and unloading processes) are adopted. Under the same strain rate, the magnitudes of temperature change during the loading and unloading process are symmetric (around 5 K). Initially, the maximum temperature change $\Delta T_{\max}$, representing the maximum temperature change between loading and unloading processes, reaches 10 K. After 4000 loading–unloading cycles, $\Delta T_{\max}$ remains stable with almost no degradation, showcasing the robust elastocaloric cyclic stability of the 1Al alloy. Furthermore, the temperature changes at the loading and unloading processes are still equal after 4000 cycles (around 5 K), which further confirms the high cyclic stability [64].

Figure 5b shows the superelasticity stress–strain curves of the 1Al alloy after 1, 1500, and 4000 loading–unloading cycles. Like the first measurement, the sample still possesses perfect superelasticity after 1500 and 4000 cycles, with no detectable residual strain. This result indicates that the stress-induced martensitic transformation still occurs in a fully reversible manner after 4000 mechanical cycles, which is consistent with the negligible degradation of $\Delta T_{\max}$ of eCE (Figure 5a). Compared with the first measurement, $\sigma_{\mathrm{cr}}$ of triggering martensitic transformation is nearly unchanged, and the maximum stress only descends by a very small value (38 MPa), further confirming the alloy’s high cyclic stability. Nevertheless, distinct from $\sigma_{\mathrm{cr}}$ and the maximum stress, $\Delta \sigma_{\mathrm{hys}}$ decreases from 72 MPa to 43 MPa after 4000 cycles, representing a reduction of 29 MPa (around 41%). The energy dissipation ΔW, defined as the area enclosed between the loading and unloading superelasticity curves, drops from 2.21 MJ m^−3^ to 1.47 MJ m^−^^3^ with a reduction of 33%. As is known, the small $\Delta \sigma_{\mathrm{hys}}$ and ΔW are beneficial for the functional stability of superelasticity, aligning well with the high cyclic stability of the 1Al alloy. Notably, the change in superelasticity stress–strain curve from the 1st to 1500th cycle is larger than that from 1500th to 4000th cycle, suggesting that $\Delta \sigma_{\mathrm{hys}}$ and ΔW tends to be stable with the loading–unloading mechanical cycle.

#### 3.3.3. Temperature Dependence

**Figure 6:**
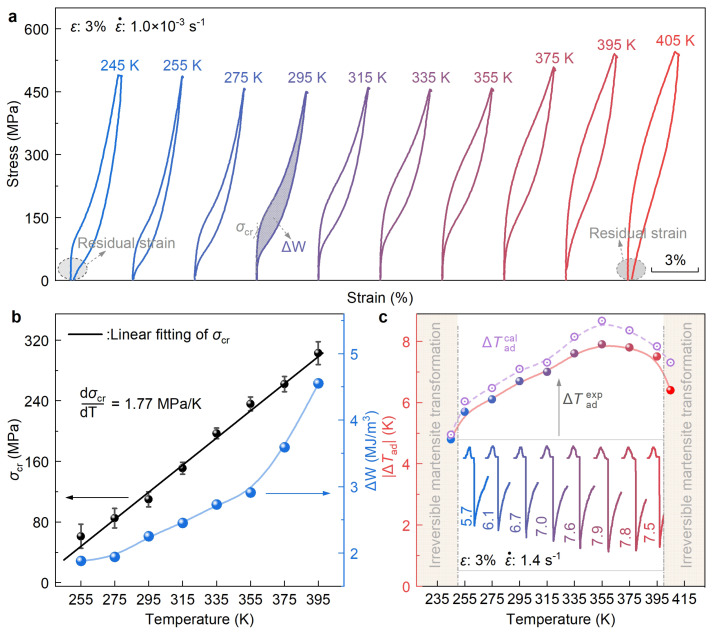
**Temperature dependence of superelasticity and elastocaloric effect of the 1Al alloy**. (**a**) Superelasticity stress–strain curves measured at various temperatures ranging from 245 K to 405 K. (**b**) Evolution of critical onset stress ($\sigma_{\mathrm{cr}}$) and energy dissipation (ΔW) of martensitic transformation. (**c**) Comparison of theoretical ($\Delta T_{\mathrm{ad}}^{\mathrm{cal}}$) and measured ($\Delta T_{\mathrm{ad}}^{\exp}$) adiabatic temperature change. The inset is the recorded temperature change profiles measured at various temperatures.

Figure 6a shows the temperature-dependent superelasticity of the 1Al alloy measured at temperatures ranging from 245 K to 405 K. Before all measurements, a ten-cycle mechanical training is performed at room temperature. Remarkably, at temperatures from 255 K to 395 K, the sample exhibits excellent superelasticity, suggesting that full reversibility of stress-induced martensitic transformation can be conserved at a temperature span of as wide as 140 K. This is greatly beneficial to realize the elastocaloric refrigeration at a wide temperature range. When the testing temperature $T_{\mathrm{Test}}$ is above 395 K or below 255 K, some irreversible residual strains are detected after the load-unloading cycle, as highlighted in Figure 6a. Thus, for the 1Al alloy, temperatures below 255 K or above 395 K are not suited for elastocaloric refrigeration. The high-temperature irreversibility (>395 K) could be attributed to the occurrence of plastic deformation mediated by dislocation slip owing to the negative temperature dependence of Peierls–Nabarro stress [65]. The low-temperature irreversibility (<255 K) should be associated with the excessive thermodynamic stability of martensite at lower temperatures [35].

Figure 6b displays the temperature dependence of $\sigma_{\mathrm{cr}}$ and energy dissipation ΔW across the martensitic transformation. With the rise in testing temperature, $\sigma_{\mathrm{cr}}$ exhibits a linear increasing trend from 61 MPa (255 K) to 303 MPa (395 K). The increase rate of $\sigma_{\mathrm{cr}}$ against temperature, i.e., $\frac{d\sigma_{\mathrm{cr}}}{dT}$, is fitted to be 1.77 MPa K^−^^1^, which is comparable to those of TiZrNbSn (1.83 MPa K^−^^1^) [33] and TiZrCrSn (1.81 MPa K^−^^1^) [35]. Similar to $\sigma_{\mathrm{cr}}$, the value of energy dissipation ΔW also gradually increases with temperature. At temperatures below 355 K, the increase rate of ΔW is relatively slow, but it rapidly accelerates beyond this temperature. This phenomenon could be attributed to the sudden increase of maximum stress at 375 K compared with that at 355 K under the constant strain of 3% (Figure 6a).

Lastly, $\Delta T_{\mathrm{ad}}$ of eCE of the 1Al alloy at different temperatures are, respectively, evaluated from both aspects of theoretical calculation and experimental measurements. For the theoretical calculation, $\Delta T_{\mathrm{ad}}^{\mathrm{cal}}$ is calculated by the isothermal transformation entropy change $\Delta S_{\sigma }$ (Equation (1)), which is estimated by the Clausius–Clapeyron relation [19] (Equation (2)).

(1) $$\begin{array}{c} \;\Delta T_{\mathrm{ad}}^{\mathrm{cal}}=\frac{\Delta S_{\sigma }\;\cdot \;T}{c_{p}} \end{array}$$

(2) $$\begin{array}{c} \;\Delta S_{\sigma }=\frac{1}{\rho }\frac{d\sigma_{\mathrm{cr}}}{dT}\Delta \varepsilon_{\mathrm{tr}} \end{array}$$

where ρ is the mass density (4.968 g cm^−^^3^), $\Delta \varepsilon_{\mathrm{tr}}$ is the transformation strain (Supplementary Materials Figure S3) and $c_{p}$ is the specific heat capacity (∼390 J kg^− 1^ K^− 1^ [33]). In Figure 6c, we compare the calculated ($\Delta T_{\mathrm{ad}}^{\mathrm{cal}}$) and measured ($\Delta T_{\mathrm{ad}}^{\exp}$) adiabatic temperature changes at different temperatures. We see that despite a slight difference in magnitude, the temperature dependence of $\Delta T_{\mathrm{ad}}^{\mathrm{cal}}$ and $\Delta T_{\mathrm{ad}}^{\exp}$ aligns well with each other, i.e., increase initially and then decrease with temperature. Remarkably, at the whole reversible temperature span of 140 K (from 255 K to 395 K), a large $\Delta T_{\mathrm{ad}}$ with a mean value of around 7 K is detected, suggesting that the present alloy possesses an excellent wide-temperature elastocaloric effect. The maximum of $\Delta T_{\mathrm{ad}}$ appears at 355 K. At this temperature, $\Delta T_{\mathrm{ad}}^{\exp}$ is measured to be −7.9 K, which is close to that of theoretical one (−8.4 K). Notably, $\Delta T_{\mathrm{ad}}$ measured at 355 K is even larger than that recorded at room temperature (−6.7 K), suggesting the 1Al alloy possesses a slightly superior eCE performance at temperatures slightly above room temperature.

## 4. Conclusions

In summary, the elastocaloric performance of TiZrNbAl shape memory alloys at both room temperature and finite temperatures ranging from 245 K to 405 K, as the first attempt, is studied. Despite the absence of thermal-induced martensitic transformation, a fully reversible superelasticity is detected in Ti-19Zr-14Nb-(1,2)Al (at.%) alloys. The alloying with Al strongly stabilizes the β austenite phase and thus surpasses the structural transformation. Compared with Ti-19Zr-14Nb-(2,3)Al alloys, Ti-19Zr-14Nb-1Al (dubbed as 1Al) possesses the lowest $\sigma_{\mathrm{cr}}$ (around 100 MPa) and a small stress hysteresis (around 70 MPa), demonstrating a substantial advantage as an elastocaloric refrigerant. For mechanical properties, the 1Al alloy exhibits excellent fracture resistance with a maximum compressive strain of 20% and compressive stress of 1.7 GPa. At room temperature, a large $\Delta T_{\mathrm{ad}}$ of −6.7 K is detected, comparable to the reported highest $\Delta T_{\mathrm{ad}}$ in the Ti-based shape memory alloy. High cyclic stability of the elastocaloric effect, with almost no degradation of $\Delta T_{\mathrm{ad}}$ after 4000 cycles, is observed. Moreover, full reversible superelasticity and a large elastocaloric effect of the 1Al alloy are consistently observed over a temperature range from 255 K to 395 K, with a temperature window as large as 140 K. At these temperatures, the mean $\Delta T_{\mathrm{ad}}$ is approximately −7.0 K, reaching a maximum value of −7.9 K at 355 K. The results of this work show a promising potential of TiZrNbAl as a low critical stress and low hysteresis elastocaloric refrigerant.

## Data Availability

The data presented in this study are available on request from the corresponding author.

## Supplementary Materials

The following supporting information can be downloaded at: https://www.mdpi.com/article/10.3390/ma17040885/s1, Figure S1: Effect of mechanical training on superelasticity; Figure S2: Room-temperature phase distribution measured by EBSD; Figure S3: Temperature dependence of transformation strain across martensitic transformation.
