# Supplementary material for: Realization of Large Low-Stress Elastocaloric Effect in TiZrNbAl Alloy
Source: Materials (Basel). 2024 Feb 14;17(4):885. doi: 10.3390/ma17040885 (PMC10889978; doi:10.3390/ma17040885)
Supplement: Supplementary file 1 [file materials-17-00885-s001.zip › materials-2865906-supplementary.pdf]

Supplementary Material to
"Realization of large low-stress elastocaloric effect in TiZrNbAl alloy"

Bang-He Lv,[1] Hua-You Xiang,[1] Shang Gao,[1] Yan-Xin Guo,[1] Jin-Han Yang,[1] Nai-Fu Zou,[2] Xiaoli Zhao,[1] Zongbin Li,[1] Bo Yang,[1] Nan Jia,[1] Hai-Le Yan,[1,*] and Liang Zuo[1]

[1]*Key Laboratory for Anisotropy and Texture of Materials (Ministry of Education), School of Material Science and Engineering, Northeastern University, Shenyang 110819, China*

[2]*Institute of Materials Science and Engineering, Shenyang Aerospace University, Liaoning, Shenyang, 110136, China*

Supplementary caption:
The Supplementary Materials contain the effect of mechanical training on superelasticity, the room-temperature phase distribution, and the temperature dependence of transformation strain across martensitic transformation of the Ti-19Zr-14Nb-1Al (at.%) alloy.

Contents:

SI: Effect of mechanical training on superelasticity.
SII: Room-temperature phase distribution measured by EBSD.
SIII: Temperature dependence of transformation strain across martensitic transformation.
SIV: Reference.

I. EFFECT OF MECHANICAL TRAINING ON SUPERELASTICITY

Fig. S1 shows the cyclic stress-strain curves of the 1Al alloy. The critical stress σ_{cr} of superelasticity decreases drastically during the first several cycles and then gradually tends to be stable. This phenomenon, known as the mechanical training effect [1], has also been observed in TiZrNbSn [2], TiNbZr [3] and TiNbMo [4] alloys. After the mechanical training, the 1Al alloy exhibits perfect superelasticity with high stability.

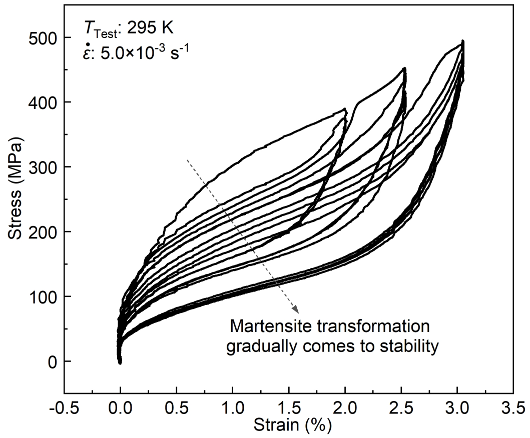

FIG. S1. **Evolution of stress-strain curves during 10-cycle mechanical training.** At the 1st-2nd, 3rd-5th and 6th-10th cycles, the applied strain gradually increases from 2.0% to 2.5% and 3%.

II. ROOM-TEMPERATURE PHASE DISTRIBUTION MEASURED BY EBSD

Fig. S2 shows the phase distribution map of the 1Al alloy measured by the EBSD technique at room temperature. Clearly, the alloy is composed of a single austenite phase at room temperature, and no martensite phase is detected.

---

* yanhaile@mail.neu.edu.cn

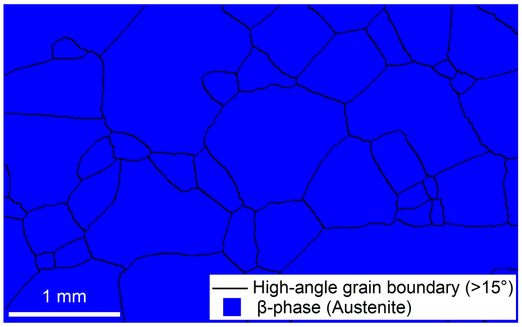

FIG. S2. **Phase distribution map of the 1Al alloy.** The blue area represents the β-phase (austenite). The black lines represent the grain boundaries with a misorientation angle larger than 15°.

III. TEMPERATURE DEPENDENCE OF TRANSFORMATION STRAIN ACROSS MARTENSITIC TRANSFORMATION

Fig. S3 shows the temperature dependence of transformation strain ($\Delta\varepsilon_{tr}$) of the 1Al alloy. $\Delta\varepsilon_{tr}$ varies slightly within the temperature range of 255 K to 355 K, reaching its maximum at 355 K. When the testing temperature is below 255 K or above 355 K, $\Delta\varepsilon_{tr}$ significantly decreases.

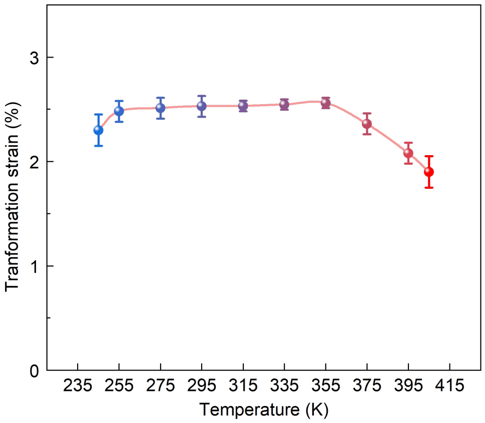

FIG. S3. **Temperature dependence of transformation strain of the 1Al alloy.**

IV. REFERENCE

[1] Feng X Q, Sun Q. Shakedown analysis of shape memory alloy structures, International Journal of Plasticity, 2007, 23(2): 183-206.
[2] Fu J, Yamamoto A, Kim H Y, et al. Novel Ti-base superelastic alloys with large recovery strain and excellent biocompatibility[J]. Acta biomaterialia, 2015, 17: 56-67.
[3] Kim J I, Kim H Y, Inamura T, et al. Effect of annealing temperature on microstructure and shape memory characteristics of Ti–22Nb–6Zr (at%) biomedical alloy[J]. Materials transactions, 2006, 47(3): 505-512.
[4] Al-Zain Y, Sato Y, Kim H Y, et al. Room temperature aging behavior of Ti–Nb–Mo-based superelastic alloys[J]. Acta materialia, 2012, 60(5): 2437-2447.
